# Chronic kidney disease predicts poor outcomes of COVID-19 patients

**DOI:** 10.1007/s11255-020-02758-7

**Published:** 2021-01-04

**Authors:** Mahmut Gok, Hakki Cetinkaya, Tugba Kandemir, Erdem Karahan, İzzet Burak Tuncer, Cengiz Bukrek, Gulizar Sahin

**Affiliations:** 1 Nephrology Department, Sultan Abdulhamid Han Training Hospital, Istanbul, Turkey; 2 Internal Medicine Department, Sultan Abdulhamid Han Training Hospital, Istanbul, Turkey

**Keywords:** Chronic kidney disease, COVID-19, Acute kidney injury, Mortality

## Abstract

**Purpose:**

The recent outbreak of COVID-19 rapidly spread worldwide. Comorbid diseases are determinants of the severity of COVID-19 infection and mortality. The aim of this study was to explore the potential association between chronic kidney disease (CKD) and the severity of COVID-19 infection.

**Methods:**

The study included 609 consecutive adult patients (male: 54.52%, mean age: 59.23 ± 15.55 years) hospitalized with the diagnosis of COVID-19 in a tertiary level hospital. Data were collected from the electronic health records of the hospital. The patients were separated into two groups: Group I included COVID-19-positive patients with CKD stage 1–2, and Group II included COVID-19-positive with CKD stage 3–5. The relationships were examined between CKD stage, laboratory parameters and mortality.

**Results:**

Significant differences were determined between the groups in respect of the inflammation parameters and the parameters used in prognosis. In Group II, statistically significantly higher rates were determined of comorbid diseases [hypertension (*p* < 0.001) and diabetes mellitus (*p* < 0.001), acute kidney injury (AKI), which was found to be associated with mortality (*p* < 0.001), and mortality (*p* < 0.001)]. In multivariate regression analysis, CKD stage 3–5, AKI, male gender, hypertension, DM and malignancy were found to be significant independent variables increasing mortality.

**Conclusion:**

The prevelance of CKD stage 3–5 on admission is associated with a high risk of in-hospital mortality in patients with COVID-19. Close follow-up can be recommended for patients with a reduced glomerular filtration rate (GFR).

## Introduction

Since the outbreak of coronavirus disease 2019 (COVID-19) in December 2019, the disease has spread rapidly across the world. COVID-19 is estimated to have a case fatality rate of 2–3%, but a wide range of mortality rates between 0.2 and 8% has been reported, most likely reflecting variable background implementation of public health interventions. The identification of factors predicting severe infection is essential to enable risk stratification, optimize allocation of hospital resources, and guide public health recommendations and interventions [1].

It has been reported that on admission, 20–51% of patients have at least one comorbidity, the most common of which are diabetes (10–20%), hypertension (10–15%) and other cardiovascular and cerebrovascular diseases (7–40%) [2–4]. Severe presentations with often fatal outcomes have consistently been associated with several comorbidities including cardiovascular disease and advanced age [5]. Given the current understanding of the weakened immune system in diabetics and individuals of advanced age, the reason is most likely immunological. It is not clear whether dialysis-dependent patients with end-stage renal disease (ESRD) are at greater risk for severe disease manifestations and poorer outcomes.

The prevalence of CKD in-patients diagnosed with COVID-19 has been reported as 1–2% [6, 7]. CKD is associated with an increased risk of both in-patient and outpatient pneumonia [8]. Moreover, pulmonary infectious mortality of patients with CKD is approximately tenfold higher than that of the general population [9]. The aim of this study was to explore the potential association between CKD and the severity of COVID-19 infection.

## Materials and methods

### Participants

All the patients enrolled in this study were diagnosed COVID-19 positive according to the guidance provided by the Turkish National Health Commission. The inclusion criteria were as follows: clinical diagnosis criteria of (1) fever or respiratory symptoms and (2) the need for clinical hospitalization (severe acute respiratory infections); and (3) the exclusion of other diseases, (4) to determine PCR testing positive in possible cases (The Republic of Turkey, Ministry of Health COVID-19 general information, epidemiology and diagnostic manual).

All of the participants were selected from 1287 COVID-19 positive patients. Pediatric and non-CKD patients were excluded from the study. After this exclusion, the study included 609 CKD patients. Clinical outcomes were monitored up to June 1, 2020, the final date of follow-up for this study. CKD was defined as the presence of kidney damage or decreased kidney function for ≥ 3 months, irrespective of the cause. CKD staging was applied with the accepted classification [10].

### Data sources

The demographic characteristics, clinical symptoms, laboratory data, and medications were extracted from electronic medical records. Laboratory data consisted of complete blood count, liver and renal function tests, and the measurement of C-reactive protein, D-dimer, ferritin, lactate dehydrogenase. The thresholds of these measures were provided by the hospital laboratory. The data were reviewed by a team of experienced physicians. Estimated glomerular filtration rate was calculated using the Chronic Kidney Disease Epidemiology Collaboration (CKD-EPI) equation [11].

The severity of COVID-19 disease was staged according to the guidelines for diagnosis and treatment of COVID-19 (trial fifth edition) published by the Chinese National Health Commission on February 4, 2020. Severe cases were defined as (1) respiratory rate > 30 breaths/min, (2) oxygen saturation < 93%, or (3) PaO_2_/FiO_2_ ratio < 300 mmHg. Critical severe cases were defined as including one of the following criteria: shock; respiratory failure requiring mechanical ventilation; combination with other organ failures; and admission to intensive care unit. Acute kidney injury (AKI) was defined as an increase in serum creatinine by 0.3 mg/dl within 48 h or a 50% increase in serum creatinine from baseline within 7 days according to the KDIGO criteria [12]. Baseline serum creatinine was defined as the serum creatinine value on admission. The date of AKI onset was defined as the earliest day of a serum creatinine change meeting the KDIGO criteria.

### Covariates

Based on a review of the literature, several covariates were selected for analysis as potential confounding variables in the regression analyses. These included age, gender, comorbidities, disease severity, and lymphocyte count.

### Statistical methods

All analyses were conducted using SPSS version 20.0 software (IBM) and Microsoft Excel. A two-tailed *p* value < 0.05 was considered statistically significant in all analyses.

All data were tested for normality of distribution. The Student’s *t* test was applied to comparisons of two groups of variables with normal distribution; one-way analysis of variance (ANOVA-post hoc: Bonferroni) was applied to the comparisons of four groups. Normally distributed continuous variables were expressed as mean ± standard deviation values and categorical variables as number and percentage and were compared using the *χ*^2^ tests. The length of stay in hospital, survival, and differences in CKD were compared between groups using the Kaplan–Meier method with log-rank testing.

Binary logistic regression analysis (Enter) was performed to test independent associations of survival with other parameters. Multinominal logistic regression analysis (Enter) was performed to test independent association of “survival and CKD” with other parameters.

## Results

We examined a total of 609 unique patients. The demographic and clinical characteristics of the patients, stratified by survival are shown in Table 1. Patients were usually middle to old age (median 59; range 21–99 years) and male gender (54.5%) was more prominent. Hypertension was the most common comorbid disease, followed by diabetes mellitus.

**Table 1:** Clinical, laboratory parameters and comorbidities of the COVID-19 patients

	All patients (*n *= 609)	Survivors (*n* = 515)	Non-survivors (*n* = 94)	*p* value
Gender (male)^a^	332 (54.52)	269 (52.23)	63 (67.02)	< 0.001
Age (years)^b^	59.23 ± 15.55	57.1 ± 14.99	70.8 ± 13.33	< 0.001
Hospitalization duration (days)^b^	9.24 ± 7.23	8.7 ± 6.45	12.2 ± 10.05	< 0.001
ICU hospitalization (*n*)^a^	121 (19.9)	27 (5.24)	94 (100)	< 0.001
*Comorbidities*				
Hypertension^a^	240 (39.41)	188 (36.5)	52 (55.32)	< 0.001
ACEI-ARB use^a^	164 (68.33)	128 (68.09)	36 (69.23)	0.87
CKD (stage 3–5) ^a^	126 (20.86)	80 (15.73)	46 (48.94)	< 0.001
Diabetes mellitus^a^	135 (22.17)	104 (20.19)	31 (32.98)	< 0.006
Malignancy^a^	23 (3.78)	14 (2.72)	9 (9.57)	< 0.001
Acute kidney injury (AKI)^a^	150 (24.63)	77 (14.95)	73 (77.66)	< 0.001
*Laboratory parameters*				
Admission creatinine (mg/dL)^b^	1.08 ± 0.55	1.04 ± 0.42	1.31 ± 0.97	< 0.001
Discharged creatinine (mg/dL)^b^	1.23 ± 1.03	0.97 ± 0.31	2.77 ± 2	< 0.001
Admission GFR (mL/min/1.73 m^2^)^b^	74.08 ± 22.79	75.73 ± 21.38	64.64 ± 27.91	< 0.001
Discharged GFR (mL/min/1.73 m^2^)^b^	74.05 ± 27.17	80.25 ± 21.19	37.35 ± 29.69	< 0.001
AST (U/L)^b^	79.45 ± 249.64	45.84 ± 46.88	265.53 ± 597.97	< 0.001
LDH (U/L)^b^	728.02 ± 763.41	570.79 ± 249.55	1598.71 ± 1610.12	< 0.001
CRP (mg/dL)^b^	96.46 ± 85.36	77.02 ± 72.47	204.11 ± 70.01	< 0.001
Ferritin (ng/mL)^b^	1155.41 ± 3789.96	543.48 ± 1304.85	4805.67 ± 8663.51	< 0.001
d-Dimer (ng/mL)^b^	2284.92 ± 4383.89	1431.06 ± 2582.02	7319.74 ± 8010.94	< 0.001
White cell count (per 10^3^/μL)^b^	5894.81 ± 8643.52	5298.56 ± 2520.25	9190.22 ± 21,064.98	< 0.001
Lymphocyte count(per 10^3^/μL)^b^	1106.13 ± 673.34	1216.03 ± 655.57	497.53 ± 385.01	< 0.001
Hemoglobin (g/dL)^b^	11.55 ± 2.08	11.95 ± 1.83	9.35 ± 2.04	< 0.001
Platelets (per 10^3^/μL)^b^	187.22 ± 78.26	194.99 ± 72.53	144.17 ± 93.85	< 0.001

The columns show the survival status of COVID-19 patients; the rows show patient clinical and laboratory parameters. Data are expressed as mean ± SD for quantitative parameters and *n* (%) for nominal parameters. (*p* < 0.05 was accepted as statistically significant)

*ICU* intensive care unit, *ACEI* angiotensin-converting enzyme inhibitor, *ARB* angiotensin receptor blocker, *CKD* chronic kidney disease, *GFR* glomerular filtration rate, *CRP* C reactive protein

^a^Chi square

^b^Independent *t* test

The differences between the survivors and non-survivors in respect of clinical, laboratory parameters and comorbidities are shown in Table 1.

In the comparison of the survivors and non-survivors, statistically significant differences were determined in respect of gender (male) (*p* < 0.001), age (*p* < 0.001), hospitalization duration (*p* < 0.001), ICU hospitalization (*p* < 0.001), hypertension (*p* < 0.001), CKD (Stage 3–5) (*p* < 0.001), diabetes mellitus (*p* < 0.006), malignancy (*p* < 0.001), acute kidney injury(AKI) (*p* < 0.001), admission creatinine (*p* < 0.001), discharged creatinine (*p* < 0.001), admission GFR (*p* < 0.001), discharged GFR (*p* < 0.001), AST (*p* < 0.001), LDH (*p* < 0.001), CRP (*p* < 0.001), ferritin (*p* < 0.001), d-dimer (*p* < 0.001), white cell count (*p* < 0.001), lymphocyte count (*p* < 0.001), hemoglobin (*p* < 0.001) and platelets (*p* < 0.001). There was no significant difference in respect of ACEI-ARB use in hypertensive COVID-19 patients (Table 1).

CKD stage 3–5 group was in minority of the patients (20.86%). These patients were older, and comorbid conditions such as diabetes and hypertension were more common among them. The differences in clinical, laboratory parameters and comorbidities after treatment for COVID-19 between patients with CKD stage 1–2 and CKD stage 3–5 are shown in Table 2.

**Table 2:** Clinical, laboratory parameters and comorbidities of the COVID-19 patients

	All patients (*n* = 609)	CKD stage 1–2 (*n* = 483)	CKD stage 3–5 (*n* = 126)	*p* value
Gender (male)^a^	332 (54.52)	267 (55.39)	65 (51.18)	0.35
Age (years)^b^	59.23 ± 15.55	55.44 ± 14.42	73.57 ± 10.61	< 0.001
Hospitalization duration (days)^b^	9.24 ± 7.23	9 ± 7.13	10.29 ± 7.55	< 0.001
ICU hospitalization (*n*)^b^	121 (19.9)	66 (14.29)	55 (43.31)	< 0.001
Mortality^a^	94 (15.44)	48 (9.94)	46 (36.5)	< 0.001
*Comorbidities*				
Hypertension^a^	240 (39.41)	154 (31.88)	86 (68.25)	< 0.001
ACEI-ARB use^a^	164 (68.33)	105 (68.09)	59 (68.6)	0.99
Diabetes mellitus^a^	135 (22.17)	93 (19.25)	42 (33.33)	< 0.001
Malignancy^a^	23 (3.78)	19 (3.94)	4 (3.17)	0.68
Acute kidney injury (AKI)^a^	150 (24.63)	81 (16.8)	69 (54.76)	< 0.001
*Laboratory parameters*				
Admission creatinine (mg/dL)^b^	1.06 ± 0.83	0.94 ± 0.21	1.64 ± 0.96	< 0.001
Discharged creatinine (mg/dL)^b^	1.23 ± 1.03	0.99 ± 0.58	2.17 ± 1.68	< 0.001
Admission GFR (mL/min/1.73 m^2^)^b^	70.75 ± 26.14	82 ± 16.97	43.03 ± 14.72	< 0.001
Discharged GFR (mL/min/1.73 m^2^)^b^	74.05 ± 27.17	83.07 ± 20.9	38.2 ± 18.04	< 0.001
AST (U/L)^b^	79.45 ± 249.64	67.88 ± 209.78	123.69 ± 361.64	0.10
LDH (U/L)^b^	728.02 ± 763.41	677.36 ± 706.51	921.83 ± 928.18	0.01
CRP (mg/dL)^b^	96.46 ± 85.36	88.96 ± 82.75	125.15 ± 89.37	< 0.001
Ferritin (ng/mL)^b^	1155.41 ± 3789.96	897.19 ± 3019.4	2165 ± 5816.63	0.02
d-Dimer (ng/mL)^b^	2284.92 ± 4383.89	1901.78 ± 3804.63	3755.69 ± 5911.3	< 0.001
White cell count (per 10^3^/μL)^b^	5894.81 ± 8643.52	5380.06 ± 2733.78	6274.72 ± 3477.65	0.01
Lymphocyte count (per 10^3^/μL)^b^	1106.13 ± 673.34	1166.55 ± 668.65	875.01 ± 642.89	< 0.001
Hemoglobin (g/dL)^ba^	11.55 ± 2.08	11.85 ± 1.97	10.43 ± 2.11	< 0.001
Platelets (per 10^3^/μL)^b^	187.22 ± 78.26	190.52 ± 79.09	174.56 ± 73.97	0.04

The columns show CKD stages of COVID-19 patients; the rows show patient clinical and laboratory parameters. Data are expressed as mean ± SD for quantitative parameters and *n* (%) for nominal parameters. (*p* < 0.05 was accepted as statistically significant)

*ICU* intensive care unit, *ACEI* angiotensin-converting enzyme inhibitor, *ARB* angiotensin receptor blocker, *CKD* chronic kidney disease, *GFR* glomerular filtration rate, *CRP* C reactive protein

^a^Chi square

^b^Independent *t* test

According to the CKD stages of the COVID-19 patients, statistically significant differences were determined in respect of age (*p* < 0.001), hospitalization time (*p* < 0.001), ICU hospitalization (*p* < 0.001), hypertension (*p* < 0.001), diabetes mellitus (*p* < 0.001), acute kidney injury(AKI) (*p* < 0.001), mortality (*p* < 0.001), LDH (*p* = 0.01), CRP (*p* < 0.001), ferritin (*p* = 0.02), d-dimer (*p* < 0.001), white cell count (*p* = 0.01), lymphocyte count (*p* < 0.001), hemoglobin (*p* < 0.001) and platelets (*p* = 0.04) levels. There was no significant difference in respect of gender (male), ACEI-ARB use, malignancy, and AST (Table 2).

Binary logistic regression analysis (Enter) showed that the risk factors for mortality were determined as CKD stage 3–5 (odds ratio [OR] 2.24; confidence interval [CI] 1.77–2.84; *p* = 0.000), male gender (OR 1.37; CI 1.09–1.73; *p* = 0.007), diabetes mellitus (OR 1.39; CI 1.1–1.77; *p* = 0.007), hypertension(OR 1.47; CI 1.18–1.83; *p* = 0.001), and malignancy (OR 1.95; CI 1.26–3,01; *p* = 0.003). ACE-ARB use (OR 1.03; CI 0.74–1.43; *p* = 0.875) did not have any significant impact on mortality.

The patients hospitalized and treated for a diagnosis of COVID-19 were separated into 4 groups according to the combinations of survival and CKD stage 3–5. These were (1) survivor-CKD stage 3–5(−), (2) survivor-CKD stage 3–5(+), (3) non-survivor-CKD stage 3–5(−), and (4) non-survivor-CKD stage 3–5(+).

Statistically significant differences were determined in respect of hypertension (*p* < 0.001), diabetes mellitus (*p* = 0.002), malignancy (*p* = 0.004), AKI (*p* < 0.001), admission creatinine (*p* < 0.001), discharged creatinine (*p* < 0.001), admission GFR (*p* < 0.001), discharged GFR (*p* < 0.001), AST (*p* < 0.001), LDH (*p* < 0.001), CRP (*p* < 0.001), ferritin (*p* < 0.001), d-dimer (*p* < 0.001), white cell count (*p* < 0.001), lymphocyte count (*p* < 0.001), hemoglobin (*p* < 0.001) and platelets (*p* < 0.001). No statistically significant difference was determined in respect of ACEI-ARB use, (*p* = 0.54) (Table 3).

**Table 3:** Clinical, laboratory parameters and comorbidities of the COVID-19 patients according to the combinations of survival and CKD stages

	Survivors and CKD (1–2) (*n* = 435)	Survivors and CKD 3–5 (*n* = 80)	Non-survivors and CKD 1–2 (*n* = 48)	Non-survivors and CKD 3–5 (*n* = 46)	*p*
*Comorbidities*					
Hypertension^a^	132 (30.34)	56 (70)	22 (45.83)	29 (63.04)	< 0.001
ACEI-ARB use^a^	92 (69.7)	36 (64.29)	13 (59.09)	22 (75.86)	0.54
Diabetes mellitus	79 (18.16)	25 (31.25)	14 (29.17)	17 (36.95)	0.002
Malignancy^a^	13 (2.99)	1 (1.25)	6 (12.5)	3 (6.52)	0.004
Acute kidney injury (AKI)^a^	46 (10.57)	31 (38.75)	35 (72.92)	38 (82.61)	< 0.001
*Laboratory parameters*					
Admission creatinine (mg/dL)^b^	0.95 ± 0.2	1.54 ± 0.81	0.83 ± 0.24	1.85 ± 1.19	< 0.001^13456^
Discharged creatinine (mg/dL)^b^	0.9 ± 0.18	1.36 ± 0.51	1.89 ± 1.51	3.8 ± 2.02	< 0.001^123456^
Admission GFR (mL/min/1.73m^2^)^b^	81.64 ± 16.89	44.03 ± 13.61	85.24 ± 17.46	41.1 ± 16.65	< 0.001^13456^
Discharged GFR (mL/min/1.73m^2^)^b^	86.39 ± 16.33	47.36 ± 11.86	52.41 ± 31.4	19.66 ± 13.67	< 0.001^12356^
AST (U/L)	45.53 ± 47.74	47.51 ± 42.17	269.94 ± 618.94	260.82 ± 581.71	< 0.001^2345^
LDH (U/L)	566.07 ± 251.72	596.09 ± 237.46	1683.6 ± 1837.44	1508.16 ± 1340.91	< 0.001^2345^
CRP (mg/dL)	75.32 ± 72.32	86.13 ± 73.01	212.29 ± 68.07	195.38 ± 71.76	< 0.001^2345^
Ferritin (ng/mL)	500.3 ± 717.88	773.77 ± 2838.44	4707.4 ± 8785.48	4913.52 ± 8635.35	< 0.001^2345^
d-Dimer (ng/mL)	1317.58 ± 2385.63	2036.28 ± 3400.83	7637.5 ± 8109.47	6994.58 ± 7991.44	< 0.001^2345^
White cell count (per 10^3^/μL)	5165.4 ± 2306.74	6010.37 ± 3374.5	7316.46 ± 4812.83	6761.36 ± 3649.15	< 0.001^23^
Lymphocyte count (per 10^3^/μL)	1240.75 ± 656.83	1083.59 ± 636.49	495.63 ± 300.71	499.56 ± 461.88	< 0.001^2345^
Hemoglobin (g/dL)	12.13 ± 1.77	11.02 ± 1.86	9.33 ± 1.96	9.38 ± 2.14	< 0.001^12345^
Platelets (per 10^3^/μL)	195.8 ± 74.74	190.65 ± 59.53	142.83 ± 99.84	145.6 ± 88.13	< 0.001^2345^

Data are expressed as mean ± SD for quantitative parameters and *n* (%) for nominal parameters

*p* < 0.05 was accepted as statistically significant

*CKD* chronic kidney disease, *ACEI* angiotensin-converting enzyme inhibitor, *ARB* angiotensin receptor blocker, *GFR* glomerular filtration rate, *CRP* C reactive protein

^a^Chi square

^b^ANOVA (post hoc: Bonferroni 1: Life and CKD 1–2 vs Life and CKD 3–5, 2: Life and CKD 1–2 vs Ex and CKD 1–2, 3: Life and CKD 1–2 vs Ex and CKD 3–5, 4: Life and CKD 3–5 vs Ex and CKD 1–2, 5: Life and CKD 3–5 vs Ex and CKD 3–5, 6: Ex and CKD 1–2 vs Ex and CKD 3–5)

**Fig. 1:**
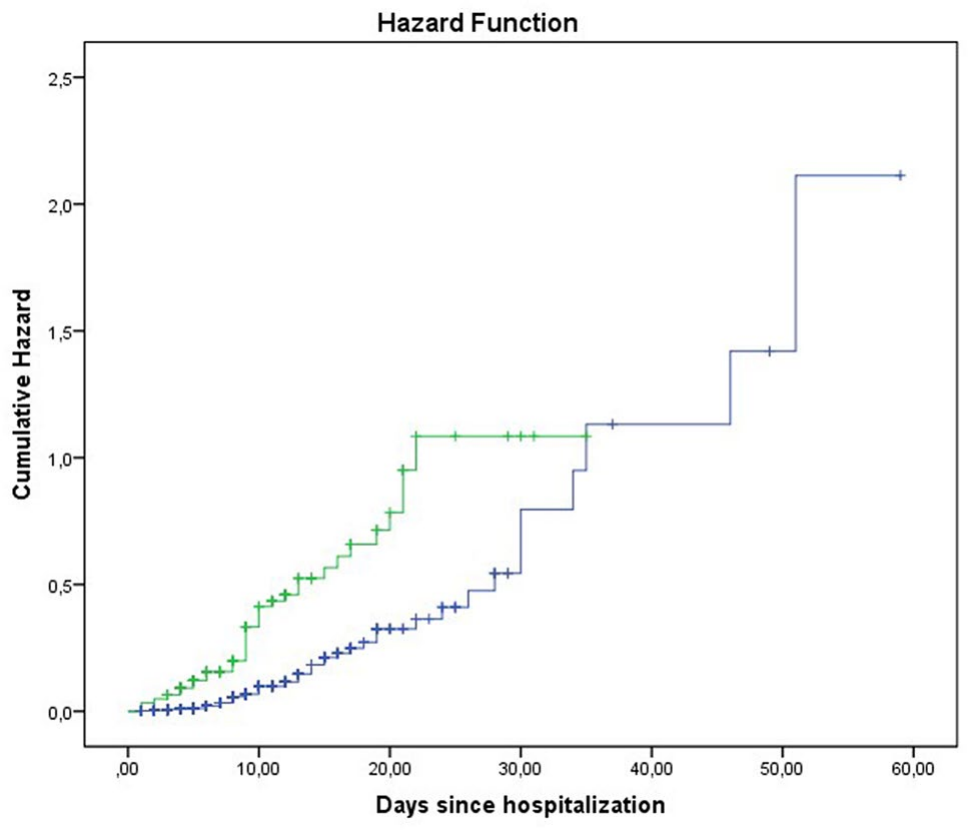
Cumulative hazard plots of the patients stratified by eGFR on admission. The green line depicts patients with CKD stage 3–5 and the blue line depicts patients with CKD stage 1–2. (In CKD stage 3–5 group hospitalization duration 24.97 ± 13.61 days (median 19 ± 2.2), in CKD Stage 1–2 group hospitalization duration 32.5 ± 2.7 days (median 30 ± 3.09). (Log rank *p* < 0.0001)

The mortality rate of patients with CKD stage 3–5 was significantly higher than that of patients with CKD stage 1–2 (Fig. 1).

As a result of the multinominal logistic regression analysis (Enter) applied, when CKD stage 3–5+ non-survivor was taken as the reference for the AKI variable, a statistically significant effect was determined of CKD stage 1–2+ survivor (OR 49.284; CI 13.49–179.96; *p* = 0.001) and CKD stage 1–2+ non-survivor (OR 15.044; CI 4.015–56.364; *p* = 0.001) and the effect of CKD stage 3–5+ non-survivor was not statistically significant (OR 1.861; CI 0.368–9.413; *p* = 0.453). No statistically significant effect was found of the other comorbidity variables according to the reference CKD stage 3–5+ non-survivor status (*p* > 0.05).

## Discussion

Previous studies have suggested that COVID-19 in people with chronic comorbidities can more easily become a critical illness or cause death [2]. CKD patients may be at greater risk of COVID-19 infection due immune system suppression, and may have poorer outcomes from COVID-19.

However, AKI induced by SARS-CoV-2 also affects patients with previous renal disorders. Studies have claimed that CKD is associated with severe disease in those infected with COVID-19 with an OR of 3 (95% CI 1.09–8.47).Based on a meta-analysis of early and preliminarily available data, CKD seems to be associated with an enhanced risk of severe COVID-19 infection. Patients with CKD should therefore be advised to take extra precautions to minimize the risk of exposure to the virus. Physicians should also be engaged in close monitoring of CKD patients with suspected COVID-19, for timely detection of signs of disease progression. Finally, the presence of CKD should be regarded as an important factor in future risk stratification models for COVID-19 [13].

Several reports in literature have documented the escalated risks of poorer clinical outcomes in patients with avian influenza, SARS-CoV and MERS-CoV infections [14–29]. The most common comorbidities associated with poorer prognosis include diabetes, hypertension, respiratory diseases, cardiac diseases, pregnancy, renal diseases and malignancy [16, 25, 28, 29].

Similarly, in the current study, hypertension, DM, and CKD were detected as comorbidities causing poor prognosis. In the meta-analysis by Henry et al., a high WBC value was used to determine the severity and mortality of the disease in addition to low lymphocyte and platelet levels, and high biochemical parameters (LDH, Ferritin, CRP, D-dimer). High mortality rates were also observed in the CKD stage 3–5 group [30].

In the current study, especially in patients with severe CKD, the detection of high acute inflammation parameters (CRP, ferritin, D-dimer) were seen to be parameter for a poor prognosis and were thought to cause more mortality in this patient group.

We report a high rate of AKI (24.63%). AKI is the result of sudden loss of kidney function and has been strongly associated with increased mortality and morbidity [31]. Current study patients with elevated serum creatinine were found to be more likely to develop AKI during hospitalization, which is consistent with a previous study of SARS [32]. To date, the published incidence of COVID-19–induced AKI is highly variable. In a case series of 116 non-critically ill Chinese patients from Wuhan, Wang L. et al. reported that only 12 (10.8%) experienced a small increase in serum creatinine or urea nitrogen within the first 48 h of hospital stay [33]. However, this has been challenged by more recent findings. AKI during hospital stay has been reported at an average incidence of 11% (8–17%), overall, with the highest ranges in the critically ill [23% (14–35%)] [34]. There has been shown to be an increased risk of AKI associated with age > 60 years, coexisting hypertension, and coronary artery disease.

The development of acute kidney injury has been observed more in those with chronic kidney disease. The current study results showed that in CKD stage 3–5 patients, the development of acute kidney damage led to a significant increase in mortality.

It is likely that the etiology of kidney disease involvement in patients with COVID-19 is multifactorial. The pathogenesis of kidney disease may be initially caused by the coronavirus entering kidney cells through the ACE2-dependent pathway with direct cytopathic effects [35].

Indirect effects on renal tissue, such as hypoxia, shock, and rhabdomyolysis, may occur through deposition of immune complexes of the viral antigen or virus-induced specific immunological effector mechanisms and subsequent virus-induced cytokines or mediators. Some patients with the 2009 H1N1 virus were reported to have mild to moderately elevated levels of serum creatine kinase [36]. Treatment of COVID-19 can also cause these negative effects on the kidneys to be eliminated. In the current study, a statistically significant difference was detected in the creatinine level at discharge and an increase in GFR values in all patients treated for COVID-19 infection, demonstrating that the infection has a significant effect on the kidneys.

While hypertension was accompanied by a significant increase in mortality in the current study, there was not seen to be any significant increase in mortality in those who used ACE inh./ARB as antihypertensive medication.

There were several limitations to this study. As urine analysis results were not available for the majority of patients, urine analysis data were not evaluated in this study. Urine findings due to kidney involvement in COVID-19 may not be detected. The follow-up duration of this study was limited to the length of hospital stay, so the COVID-19 effects on long-term outcomes could not be evaluated. A further limitation was that no formal power analysis was performed to determine the sample size. However, all eligible hospitalized patients were recruited. As this study was performed in a tertiary level hospital, it is possible that more severe patients were included. Finally, it may be difficult to generalize the results internationally as other countries have adopted different treatment guidelines according to local regulations and the availability of health resources.

In conclusion, CKD stage 3–5 appears to lead to a poor prognosis and increased mortality for patients with COVID-19 infection. After adjustment for confounding variables, kidney disease on admission and AKI during hospitalization were determined to be associated with an increased risk of mortality. Physicians should increase their awareness of kidney disease in hospitalized patients with COVID-19. Early detection and effective intervention of kidney involvement may help to reduce the mortality rates of patients with COVID-19.

## Data Availability

The datasets generated during and/or analyzed during the current study are available from the corresponding author on reasonable request.
